# Stunted at 10 Years. Linear Growth Trajectories and Stunting from Birth to Pre-Adolescence in a Rural Bangladeshi Cohort

**DOI:** 10.1371/journal.pone.0149700

**Published:** 2016-03-02

**Authors:** Pernilla Svefors, Anisur Rahman, Eva-Charlotte Ekström, Ashraful Islam Khan, Emma Lindström, Lars Åke Persson, Katarina Ekholm Selling

**Affiliations:** 1 International Maternal and Child Health, Department of Women’s and Children’s Health, Uppsala University, Uppsala, Sweden; 2 International Centre for Diarrhoeal Disease Research, Bangladesh (iccdr,b), Dhaka, Bangladesh; Institute for Health & the Environment, UNITED STATES

## Abstract

**Background:**

Few studies in low-income settings analyse linear growth trajectories from foetal life to pre-adolescence. The aim of this study is to describe linear growth and stunting from birth to 10 years in rural Bangladesh and to analyse whether maternal and environmental determinants at conception are associated with linear growth throughout childhood and stunting at 10 years.

**Methods and Findings:**

Pregnant women participating in the MINIMat trial were identified in early pregnancy and a birth cohort (n = 1054) was followed with 19 growth measurements from birth to 10 years. Analyses of baseline predictors and mean height-for-age Z-scores (HAZ) over time were modelled using GLMM. Logistic regression analysis was used to investigate the associations between baseline predictors and stunting (HAZ<-2) at 10 years. HAZ decreased to 2 years, followed by an increase up to 10 years, while the average height-for-age difference in cm (HAD) to the WHO reference median continued to increase up to 10 years. Prevalence of stunting was highest at 2 years (50%) decreasing to 29% at 10 years. Maternal height, maternal educational level and season of conception were all independent predictors of HAZ from birth to pre-adolescence (p<0.001) and stunting at 10 years. The highest probability to be stunted at 10 years was for children born by short mothers (<147.5 cm) (OR_adj_ 2.93, 95% CI: 2.06–4.20), mothers with no education (OR_adj_ 1.74, 95% CI 1.17–2.81) or those conceived in the pre-monsoon season (OR_adj_ 1.94, 95% CI 1.37–2.77).

**Conclusions:**

Height growth trajectories and prevalence of stunting in pre-adolescence showed strong intergenerational associations, social differentials, and environmental influence from foetal life. Targeting women before and during pregnancy is needed for the prevention of impaired child growth.

## Introduction

Child undernutrition remains one of the main global health challenges in low and middle-income countries (LMIC) [1]. Stunting of linear growth (low height for age) is currently the most dominant form of child undernutrition and is estimated to affect over 165 million children globally before the age of five years [1].Stunting, defined as an attained height below -2 standard deviations (SD) of the WHO growth reference median, indicates a failure to reach one’s potential for linear growth and is an acknowledged way of representing child health inequalities[2,3]. Stunting reflects a variety of environmental conditions influencing child growth including circumstances that lead to intrauterine growth restriction [4,5], the household socio-economic conditions and education levels of parents[6–8], inadequate nutrition of the mother and child and frequent infections [9–11].

Early linear growth retardation is associated with future negative health consequences, such as impaired cognitive development, reduced economic productivity in adulthood, unfavourable maternal reproductive outcomes and a risk of development of non-communicable diseases [12]. Understanding child growth patterns is thus critical in order to develop appropriate interventions to reduce stunting and its short- and long-term consequences for individuals and society. Growth faltering is documented to be initiated early [13] and most pronounced during the first 24 months of life [14]. Hence the concept of the first 1000 days, starting at conception, has been developed as the most important period for nutritional interventions.

Although the dynamics and timing of early growth faltering in low- and middle-income countries are well documented [14,15] most studies are based on cross-sectional surveys and focus on children up to the age of five years. Relatively few cohort studies analyse linear growth from birth and up to adolescence, limiting what conclusions can be drawn regarding child growth trajectories, stunting and the associations with the child’s environment. In addition, lately the use of absolute height differences from a reference median (HAD) has been presented as an alternative indicator of linear growth retardation, reportedly it better reflects the potential accumulation of growth deficit over time [16–18].

In this paper we aim to describe linear growth trajectories, using both height for age scores and absolute height difference from reference median, and the occurrence of stunting from birth to 10 years of age in a rural Bangladeshi setting. We also analyse whether important stunting determinants present from early life, representing maternal malnutrition (maternal height), social differentials (maternal education level), and early environment (season of conception) continue to be associated with linear growth trajectories throughout childhood and with stunting at ten years of age.

## Methods

### Participants, Study Design and Setting

Children of mothers participating in the MINIMat trial (Maternal and Infant Nutrition Interventions in Matlab, isrctn.org identifier: ISRCTN16581394) were followed from birth to 10 years of age. MINIMat was a factorial randomized trial primarily evaluating the effect of an early invitation to prenatal food supplementation (vs. usual timing) combined with multiple micronutrient supplementation (vs. usual program iron-folate) to pregnant women and the effect on anaemia in pregnancy, birth weight and infant survival. The reported main effects of the interventions so far are improved infant and under five survival and reduced stunting at 4.5 years [19,20]. The trial was carried out in Matlab, Bangladesh, a rural sub-district 57 km south- east of the capital Dhaka, in a setting where a widespread child and maternal undernutrition still prevail. In the area a Health and Demographic Surveillance System, run by the International Center for Diarrhoeal Disease Research, Bangladesh (iccdr,b) has been in place since the mid-1960s. Community health workers (CHWs) collect data on demographic and selected health information through monthly household visits. The surveillance system covers a population of about 220,000 in more than 140 villages. The use of a unique identification system allows tracking over time and across studies and databases. The MINIMat trial recruited pregnant women within the icddr,b service area from November 2001 to October 2003. If a woman reported to a CHW that her menstruation was delayed more than 14 days she was offered a pregnancy test and her date for the last menstrual period (LMP) was recorded. Dating based on ultrasound examination was performed if LMP date was missing. In total 4436 pregnant women participated, giving birth to 3625 live born infants. The children of mothers participating in the initial prenatal intervention study have so far been followed from birth to 10 years. For the 10-year follow-up 1663 children born from April 2002 to June 2003 were invited to participate, representing a one calendar year birth cohort.

### Socioeconomic and Anthropometric Measurements

Community health research workers with at least ten years of education interviewed the mothers in their homes at enrolment. They used structured questionnaires that included socioeconomic characteristics (SES), parental education and pregnancy history.

Home visits were followed by clinical visits at local sub-centers run by the icddr,b in the Matlab area. Maternal weight and height were measured at around 8 weeks of gestation using an electronic scale (Uniscale; SECA), with a precision of 0.10 kg and a stadiometer to the nearest 0.1 cm. The children’s weight and length/height were measured at birth and after that every month up to one year and then every three months up to 24 months and then again at 4.5 and 10 years. Most birth anthropometry was measured within 72 h of birth. However, measurements were made even if the new-borns were reached after 72 hours. Measurements taken from 24 hours up to 30 days after birth were adjusted using an SD score transformation, assuming that infants remain in the same relative position in the anthropometric distribution during this period [21]. The recumbent length at birth and during infancy (until 1.5 years) was measured with a locally manufactured, collapsible length board with a precision of 0.1 cm. A SECA electronic or beam scales (UNICEF Uniscale; SECA Gmbh & Co, Hamburg, Germany), with a precision of 0.01 kg was used when measuring birth weight. At follow-ups height was measured to the nearest 0.1 cm, using a freestanding stadiometer. Puberty assessment was done in girls by evaluating breast development according to Tanner staging. The children were barefoot in light clothing when measurements were taken. A team consisting of nurses, a medical doctor and a laboratory technician, assisted by trained field staff, conducted all measurements. Each measurement was taken at a similar time of the day and refresher training on data collection methods including standardization of anthropometric measurements was conducted periodically.

### Variables Included in Analyses

*The socioeconomic characteristics* of the households were determined using a continuous household asset score previously generated in this population including data on land ownership, the construction materials of house walls, ownership of household assets etc. [22]. Tertiles of asset scores were then calculated from the initial sample of 4436 women enrolled in the MINIMat study. Age and parity were categorized into three and four groups, respectively (age: <20 years, 20–30 years and >30 years; parity: first child, second child, third child, fourth or more child). *Maternal height* reflects the mother’s nutritional status accumulated through nutritional, social and environmental exposures and is a convenient indicator for assessing intergenerational associations. Tertiles of mothers’ height were created from the initial sample of 4436 women (cut offs at 147.5 and 152 cm, respectively). Height data were missing from two mothers, and these were excluded from analysis. *Maternal education* has been proven to be a strong determinant of stunting and is associated with caregiving behaviour, feeding practices, and age at first pregnancy, etc. [8,23,24]. Three categories were created based on years of formal education: no formal education, 1–5 years (primary school) and more than 5 years of education. *Season of conception* reflects the environmental exposures of the foetus and the mother. The variable was calculated based on LMP date and categorized after Bangladesh’s three distinct seasons; the hot pre-monsoon season from March through May, the rainy monsoon season from June through October and the cool and dry winter from November through February [25].

To characterize growth, both *height for age z-scores* (HAZ) and the *difference in height in cm* (HAD) from reference median were calculated. HAZ was calculated using the program WHOAnthro from birth to 4,5 years and AnthroPlus at ten years of age using WHO growth references for 0–5 and 5–19 years respectively [26,27]. Using the same references HAD was calculated as the difference between the measured height and the median age- and sex-specific height obtained from the reference data.

### Ethics

Written informed consent was obtained from all parents of participating children (separately for the original trial and the two follow-ups). The Ethical Review Committee at the International Centre for Diarrhoeal Disease Research, Bangladesh and the Regional Ethical Review Board in Uppsala, Sweden approved the study (separately for the original trial and the two follow-ups).

### Statistical Analysis

An initial dataset was selected with those having height measurements from birth, 24 months and 4.5 and 10 years. Data were linearly interpolated if one or maximum two consecutive measurements were missing in the age interval between birth and 2 years of age. For example, if the 4-month height measurement was missing, it was imputed by linear interpolation of the 3- and 5-month heights. If two consecutive values were missing, the values were imputed from the weighted average of the previous and subsequent measurements.

Generalized linear mixed models (GLMM) were used to model repeated measurements on the continuous outcome HAZ over the 19 different time points from birth to pre-adolescence. In the models Time (stating the 19 time points) was treated as nested under the random factor ID (the unique identifier for each child), thus allowing for different intercepts and slopes on the HAZ scores over time for each child. We found that the trend of HAZ over time was best explained by including a second order polynomial (Time^2^) in the models. As the time points were not equally spaced, the correlation structure of the repeated measurements was modelled using an autocorrelation structure of order 1, with a continuous time covariate (CAR)[28]. In the crude analyses the fixed factors, maternal height in tertiles, season of conception and maternal educational level were included one by one. They were also included simultaneously in one model, and additional adjustments were made for maternal parity, maternal socio-economic status and maternal age at enrolment. In addition, binary logistic regression analysis was used to quantify the relationship between maternal height in tertiles, the season of conception and maternal educational level at birth and the child’s probability of being stunted at 10 years of age. Both crude and adjusted odds ratios (ORs) were computed. All analyses were performed in R [28]. The longitudinal GLMM was performed using the nlme package [29].

## Results

**Fig 1:**
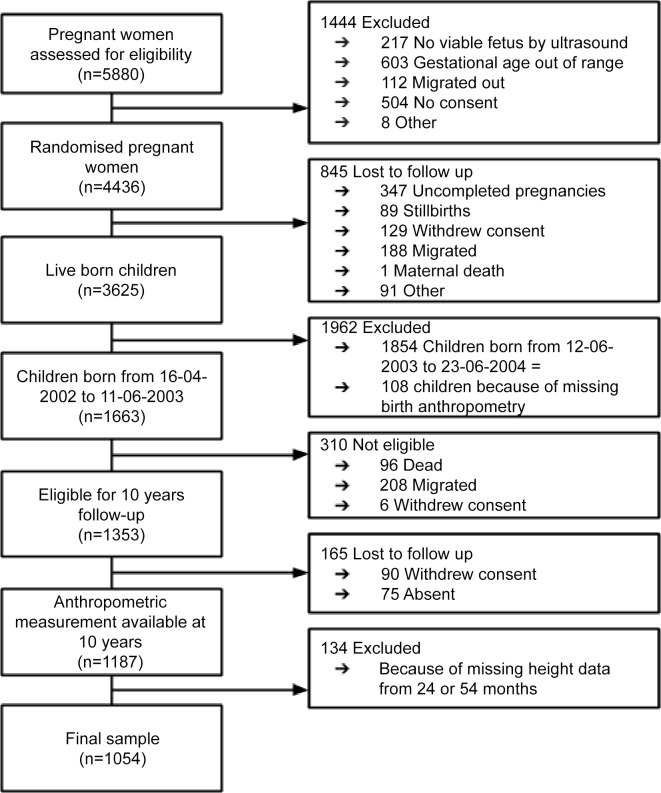
Flow Chart of children participating in the MINIMat trial from birth to 10 years of age.

There were 3625 live born children by the 4436 women enrolled into the MINIMat trial. Losses before measurement of birth anthropometry included early foetal loss (n = 347), stillbirth (n = 89), outmigration (n = 188), and refusal to participate (n = 129). Out of the one-year birth cohort born from April 2002 to June 2003 selected for the 10-year follow-up 108 had no birth anthropometry and were excluded from analysis. Losses from follow-up after birth anthropometry up to 10 years of age included outmigration (n = 208), refusal to participate (n = 6) and death (n = 96). Of the 1187 children with anthropometry from birth and 10 years 134 children were excluded because of no height measurement from either 24 or 54 months resulting in 1054 children available for longitudinal analysis (Fig 1). The randomized interventions were not associated with any of the determinants of growth included in the analysis. In the non-analysed group there were a slightly higher percentage mothers with more than 6 years of education and mothers belonging to the lowest socioeconomic tertile (p<0.05) (S1 Table). For all other characteristics there were no significant differences of mothers or children between the analysed and non-analysed group Baseline characteristics of the mothers at 8 weeks of gestation and children at birth are stated in Table 1. The participating mothers had an average age of 26 years (SD 5.90) at recruitment and an average height of 150 cm (SD 5.13). More than one-third of the women had no formal education, one-third had a BMI below 18.5 and 15% were shorter than 145 cm. The sample of children comprised of an almost equal proportion of girls and boys (49 and 51%, respectively). The average birth length and weight were 47.8 cm (SD 2.19) and 2676 grams (SD 400), respectively. More than half of the children (62%) were born small for gestational age (SGA), 32% had low birth weight (LBW) and 8% were born before 37 weeks of gestation (preterm). At ten years of age, according to Tanner breast development staging, 89% of the girls had not yet entered into puberty (stage 0 or 1) and 11% of the girls had reached stage 2 or more.

**Table 1:** Baseline characteristics of mothers at 8 week of gestation and children at birth participating in the MINIMat trial, Bangladesh.

CHARACTERISTICS	n	%
Mothers		
Age		
<20	148/1054	14.0
20–29	595/1054	56.5
≥30	311/1054	29.5
BMI at 8 week		
<18.5	325/1049	31.0
≥18.5	724/1049	69.0
Height		
Shortest (<147.5 cm)	356/1052	33.8
Average (≥147.5 <152 cm)	359/1052	34.1
Tallest (≥152 cm)	337/1052	32.0
Education		
No Education	415/1054	39.4
1–5 years	244/1054	23.1
> 5 years	395/1054	37.5
SES tertiles		
Lowest	280/1054	26.6
Middle	355/1054	33.7
Highest	419/1054	39.8
Children		
Sex		
Girl	513/1054	48.7
Boy	541/1054	51.3
Season of conception		
Winter (Nov-Feb)	353/1054	33.5
Pre-monsoon (Mars-May)	311/1054	29.5
Monsoon (Jun-Oct)	390/1054	37.0
SGA ^1^	652/1054	61,9
LBW^2^	339/1053	32.2
Stunted^3^	170/1054	16.1
Preterm ^4^	86/1054	8.2

^**1**^Small for gestational age (Birth weight below reference for gestational week at birth [30]

^**2**^Low Birth Weight (<2500 g)

^**3**^Below minus 2 SD from WHO growth reference median

^**4**^Born before 37 weeks of gestation

### Longitudinal Growth among Boys and Girls from Birth to 10 years of Age

**Fig 2:**
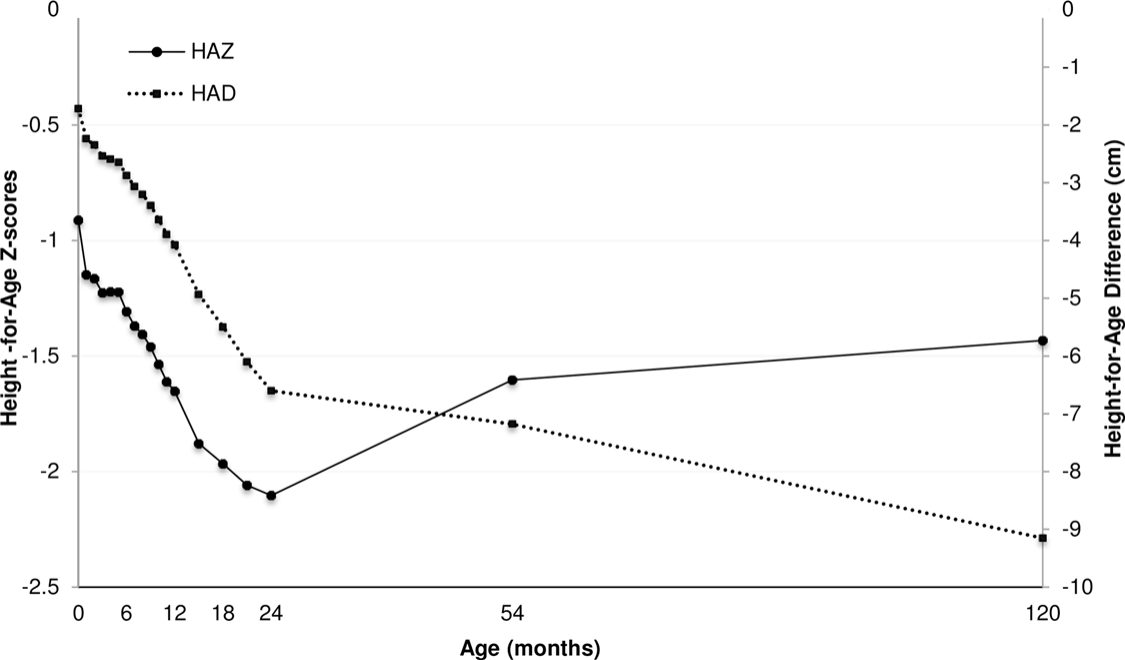
Mean HAZ and HAD scores of children participating in the MINIMat trial from birth to 10 years. N = 1054.

Height for age scores (HAZ) and the difference in height in cm from reference median (HAD) from birth up to 10 years are shown in Fig 2 and S2 Table. Mean HAZ at birth was low (– 0.91), and this measurement declined rapidly up to two years of age (mean HAZ at 2 years -2.10). From 2 to 10 years of age, HAZ increased (mean HAZ at 10 years -1.43). The difference in height in cm from reference (HAD) showed a similar declining pattern up to two years of age, but continued to increase beyond the age of 2 years.

**Fig 3:**
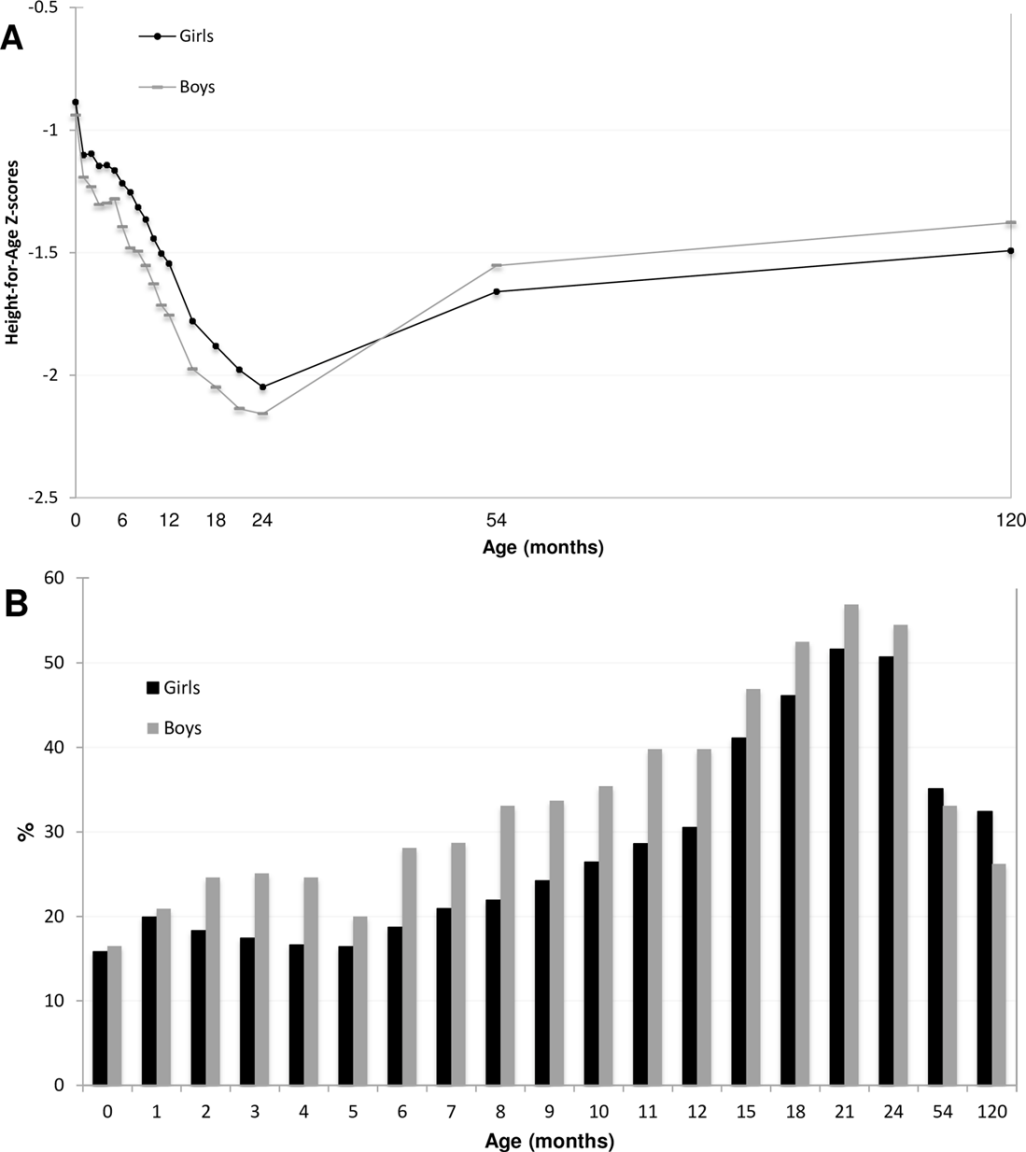
**A) Mean HAZ scores of girls and boys participating in the MINIMat trial from birth to 10 years of age.** N = 1054. **B) Stunting prevalence of girls and boys from birth to 10 years of age.** Note: the scale has been adjusted to enhance distinguishability.

The prevalence of stunting at birth was 16% and increasing to 54% at 2 years of age (S3 Table). By 4.5 years the prevalence had decreased to 34% and by 10 years to 29%. Height for age scores and stunting prevalence of girls and boys are presented in Fig 3. There was a tendency of greater growth deficits among boys during infancy and girls at 4.5 and 10 years of age (Fig 3) (Tables 2 and 3). At 6 and 12 months of age boys had a higher prevalence of stunting than girls (p-value <0.001). Later in childhood the pattern was reversed; at 10 years girls had a higher prevalence of stunting than boys (girls 32.4% and boys 26.2%, p = 0.029).

**Table 2:** Maternal height, maternal educational level and season of conception at birth in relation to the children’s HAZ scores over time.

	Crude analyses^1^			Adjusted analyses I^2^			Adjusted analyses II^3^		
	Estimate	95% CI	P	Estimate	95% CI	P	Estimate	95% CI	P
**Maternal height**									
Tallest (>152 cm)	Ref.			Ref.			Ref.		
Average (≥147.5–152 cm)	-0.38	-0.50 to -0.26	<0.001	-0.33	-0.46 to -0.21	<0.001	-0.31	-0.43 to -0.19	<0.001
Shortest (<147.5 cm)	-0.67	-0.80 to -0.54	<0.001	-0.61	-0.74 to -0.49	<0.001	-0.59	-0.71 to -0.46	<0.001
**Maternal education**									
> 5 years	Ref.			Ref.			Ref.		
1–5 years	-0.39	****	<0.001	-0.34	-0.47 to -0.21	<0.001	-0.24	-0.38 to -0.10	<0.001
No education	-0.51	****	<0.001	-0.42	-0.54 to -0.31	<0.001	-0.25	-0.39 to -0.11	<0.001
**Season of conception**									
Winter (Nov-Feb)	Ref.			Ref.			Ref.		
Pre-monsoon (Mar-May)	-0.28	-0.41 to -0.14	<0.001	-0.23	-0.35 to -0.10	<0.001	-0.21	-0.33 to -0.09	<0.001
Monsoon (Jun-Oct)	-0.08	-0.21 to 0.04	0.196	-0.07	-0.19 to 0.05	0.246	-0.07	-0.18 to 0.05	0.267

The MINIMat trial Bangladesh, N = 1054.

^**1**^ GLMM on repeated measurements on HAZ over time, allowing for different intercepts and slopes for each child. Variables in table modeled separately.

^**2**^ GLMM on repeated measurements on HAZ over time, allowing for different intercepts and slopes for each child. Variables in table modeled simultaneously.

^**3**^ GLMM on repeated measurements on HAZ over time, allowing for different intercepts and slopes for each child. Variables in table modeled simultaneously, and additional adjustments were made for maternal parity, age, and socio-economic status when giving birth.

**** Cis do not converge by neither ml nor reml estimation in using the lnme package in r (28). Thus, only p-values are represented.

**Table 3:** Maternal height, maternal educational level and season of conception at birth in relation to the children’s probability to be stunted at 10 years of age.

	Crude analyses^1^			Adjusted analyses I^2^			Adjusted analyses II^3^		
	OR	95% CI	P	OR	95% CI	P	OR	95% CI	P
**Maternal height**									
Tallest (>152 cm)	Ref.			Ref.			Ref.		
Average (≥147.5–152 cm)	1.89	1.33–2.70	<0.001	1.78	1.24–2.56	0.002	1.74	1.21–2.52	0.003
Shortest (<147.5 cm)	3.20	2.27–4.55	<0.001	3.00	2.12–4.30	<0.001	2.93	2.06–4.20	<0.001
**Maternal education**									
> 5 years	Ref.			Ref.			Ref.		
1–5 years	1.91	1.32–2.76	<0.001	1.84	1.26–2.68	0.001	1.68	0.94–2.13	0.013
No education	2.35	1.71–3.24	<0.001	2.09	1.51–2.91	<0.001	1.74	1.17–2.81	0.007
**Season of conception**									
Winter (Nov-Feb)	Ref.			Ref.			Ref.		
Pre-monsoon (Mar-May)	2.08	1.49–2.92	<0.001	1.97	1.39–2.80	<0.001	1.94	1.37–2.77	<0.001
Monsoon (Jun-Oct)	1.29	0.92–1.80	0.138	1.25	0.89–1.77	0.198	1.26	0.89–1.78	0.188

The MINIMat trial Bangladesh, N = 1054.

^**1**^ Binary logistic regression analysis. Variables in table modelled separately.

^**2**^Binary logistic regression analysis. Variables in table modelled simultaneously.

^3^Binary logistic regression analysis. variables in table modelled simultaneously, and additional adjustments were made for maternal parity, age, and socio-economic status when giving birth

### Linear Growth by Maternal Height, Education and Season at Conception

**Fig 4:**
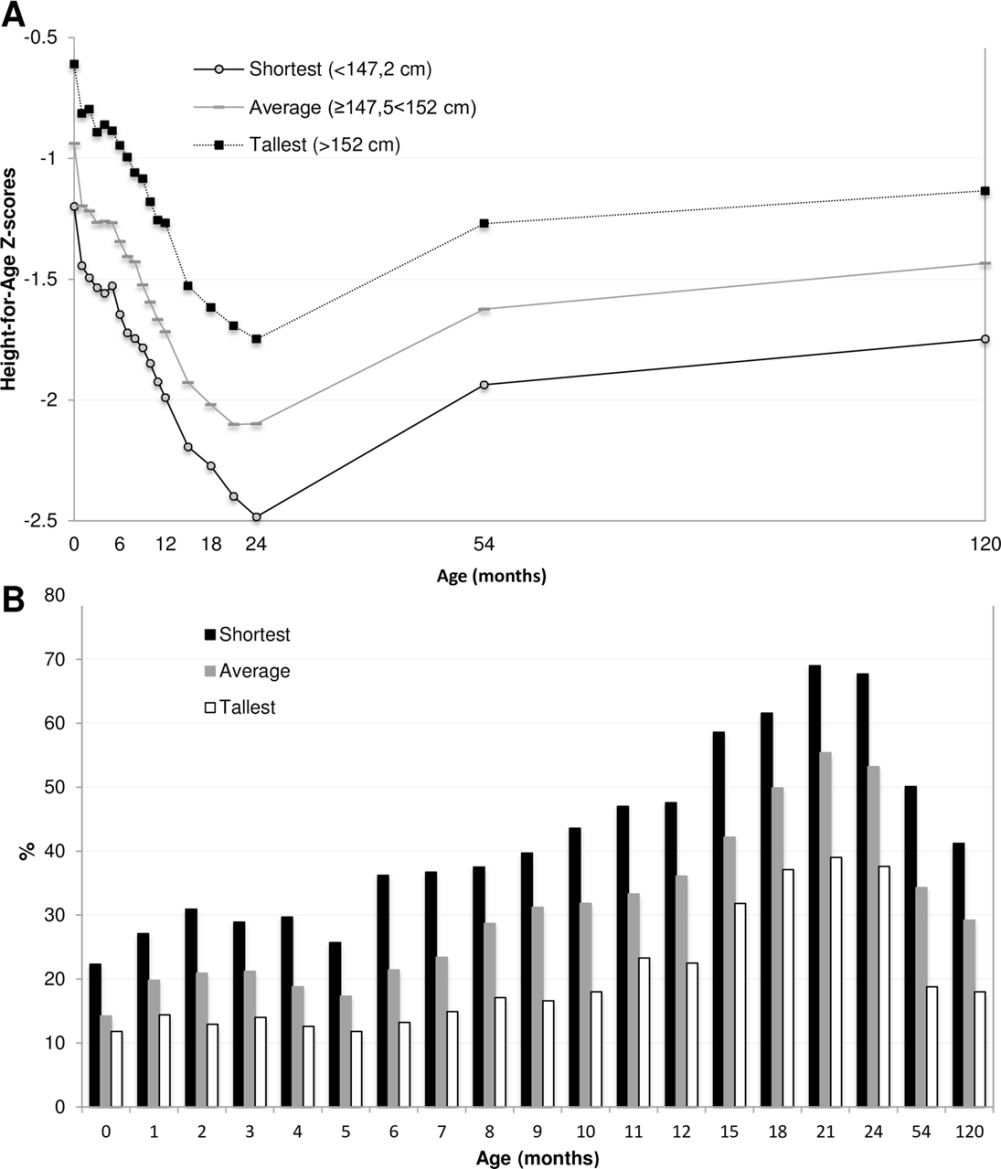
**A) Mean HAZ scores stratified for tertiles of Maternal Height from birth to 10 years of age in children participating in the MINIMat trial.** N = 1052. **B) Stunting prevalence stratified for tertiles of Maternal Height from birth to 10 years of age**. Note: the scale has been adjusted to enhance distinguishability.

**Fig 5:**
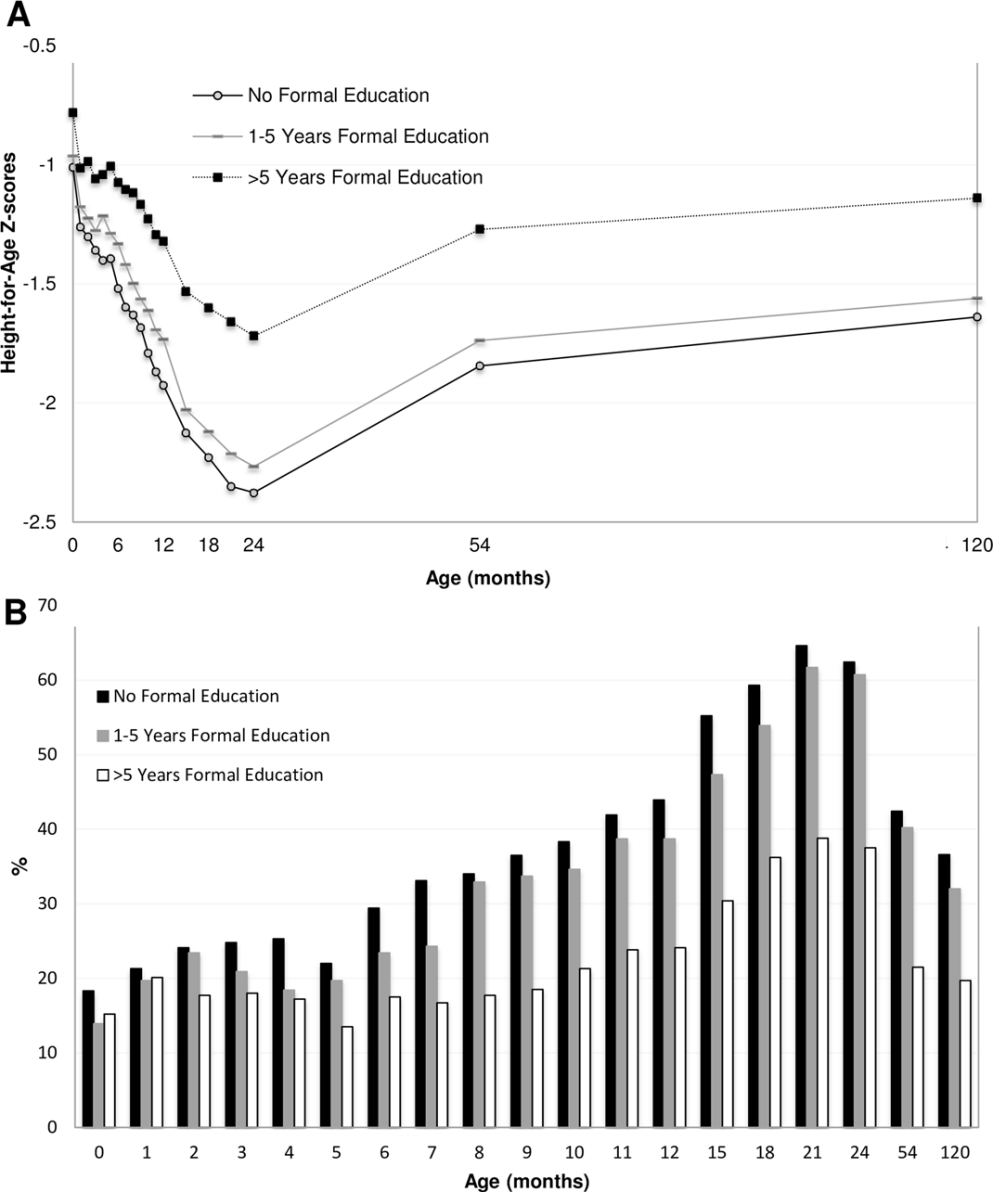
Mean HAZ scores stratified for tertiles of Maternal Education Level from birth to 10 years of age in children participating in the MINIMat trial. N = 1054. **B) Stunting prevalence stratified for tertiles of Maternal Education Level from birth to 10 years of age.** Note: the scale has been adjusted to enhance distinguishability.

**Fig 6:**
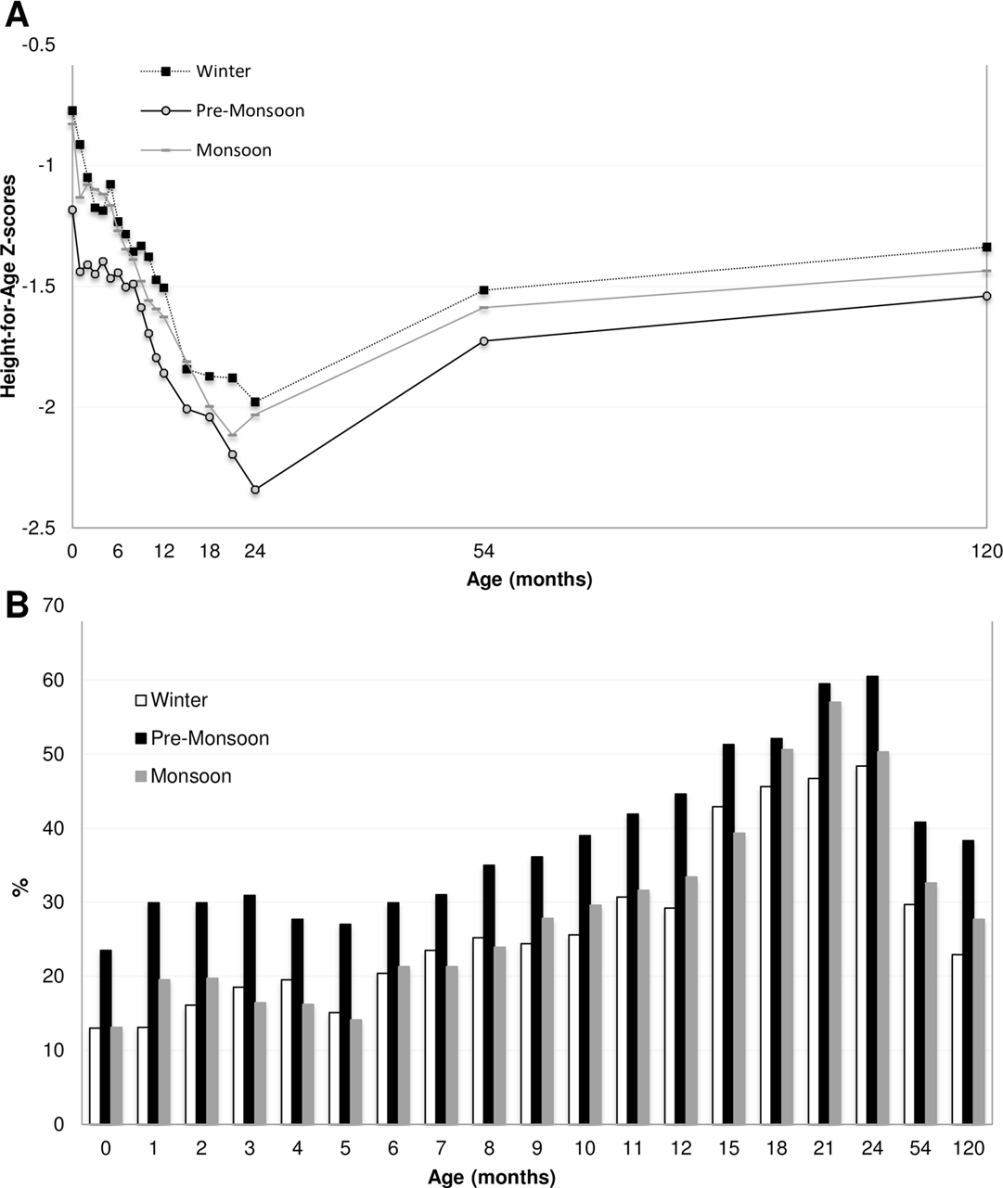
**A) Mean HAZ scores stratified for Season of Conception from birth to 10 years of age in children participating in the MINIMat trial.** N = 1054. **B) Stunting prevalence stratified for Season of Conception from birth to 10 years of age.** Note: the scale has been adjusted to enhance distinguishability.

HAZ scores stratified for mother’s height in tertiles, presented in Fig 4, displayed a parallel pattern across the studied age period, with the lowest HAZ scores in children with the shortest mothers (i.e. those shorter than 147.5 cm). The longitudinal growth analysis supported this (Table 2); children born to the shortest mothers had a mean HAZ score of -0.59_adj_ (95% CI_adj_: -0.71 to -0.46) lower than children to the tallest mothers. Children born to the shortest mothers also had a higher prevalence of stunting at all ages (Fig 4) and almost three times higher probability to be stunted at 10 years as compared to tall mothers (OR_adj_ 2.93, 95% CI: 2.06–4.20, Table 3). A similar pattern for height for age scores and stunting prevalence was present for mother’s education levels (Fig 5). Children born by mothers with no education had the lowest HAZ scores (-0.25_adj_, 95% CI_adj_: -0.39 to -0.11, Table 2) and the highest probability to be stunted at 10 years of age (OR_adj_ 1.74, 95% CI 1.17–2.81, Table 3) as compared to children born by mothers with more than 5 years of education. HAZ scores and stunting prevalence stratified for season of conception are presented in Fig 6. Children conceived in the pre-monsoon season had significantly lower HAZ scores (-0.21_adj_, 95% CI_adj_: -0.33 to -0.09, Table 2) and higher probability to be stunted at 10 years of age (OR_adj_ 1.94, 95% CI 1.37–2.77, Table 3) compared to children conceived in winter. However, we found no statistical evidence of a difference in HAZ in children who were conceived in monsoon as compared to winter.

## Discussion

In this study, birth cohort data from rural Bangladesh was used to characterize growth trajectories and stunting patterns of children from birth to ten years of age with special emphasis on whether well-known risk factors present at the time of conception continued to be associated with childhood growth and the risk of stunting at ten.

The linear growth patterns, characterized by HAZ and HAD, were consistent with results from earlier studies from LMICs [14,17]. HAZ scores, decreased from birth to 2 years of age while growth faltering expressed as a difference to reference median (HAD) continuously increased up to 10 years. Prevalence of stunting increased up to 2 years of age followed by a decrease. We observed strong intergenerational associations (short mothers and offspring stunting), associations between mother’s education level and short children and environmental influence from early life exposure (season of conception) on childhood growth and stunting at 10 years of age.

Being implemented in an excellent research infrastructure the MINIMat cohort fulfils the prerequisites for obtaining high-quality longitudinal data. The equipment was frequently controlled and experienced study nurses, who received repeated training including anthropometric standardization exercises, supervised by senior medical doctors carried out the data collection. From 2 to 10 years there were only two measurements, potentially missing the development in that interval. However, it is reasonable to believe that the main growth patterns remained relatively constant across these ages. The present paper focuses on the description of linear growth trajectories and the prevalence of stunting up to preadolescence, and analyses of the associations with relatively well-known determinants of linear growth that are present from the time of conception. However, there is a wide range of other factors that over this age span may promote or impair linear growth. Despite this complexity of contributing causes to linear growth restriction, it is of value to increase the understanding as to what extent early biological, social and environmental factors are associated with linear growth from conception to pre-puberty. The study was conducted in rural Bangladesh in a low socioeconomic setting, where undernutrition in early life is widespread, which makes the results relevant for other areas in Bangladesh, neighbouring countries in South Asia as well as for other low-income countries struggling with childhood stunting.

A comparison of childhood growth patterns in 54 low- and middle-income countries demonstrated that the main growth faltering took place from 3 to 18–24 months of age with modest recovery through mid-childhood [14]. This notion of little or no recovery in linear growth after 24 months of age has been questioned by various longitudinal studies analysing linear growth beyond infancy showing increasing HAZ and decreasing prevalence of stunting across childhood and adolescence [31–36]. The increases in HAZ from the nadir at 24 months can reflect a decreasing vulnerability to exposure of risk factors and the potential of catch up growth. However, recent studies have shown that increases in height for age scores and recovery from stunting over time may coexist with further increase in absolute height deficits as compared to the reference median [16,17]. These seemingly contradictory growth patterns were also observed in our study. The discrepancies in height for age scores and absolute height deficits reflect the increasing variation in normal growth over age but also raises questions concerning the best representation of linear growth in relation to future health and developmental outcomes. Yet, substantial research links stunting to future adverse outcomes and a decrease in stunting prevalence is hence desirable. Stunting is defined by height-for-age scores [2] and so far there is no alternative definition or threshold based upon absolute height deficits. Still, results from our and other studies demonstrating variations in HAZ and HAD beyond the age of two, regardless of the interpretation, imply that growth continues to be malleable past infancy [37]. This does not challenge the focus on the importance of the first 1000 days but have implications for additional interventions improving the nutritional status of older children.

Linear growth trajectories and stunting prevalence differed between girls and boys. Boys showed greater growth faltering during early infancy whereas girls had a higher prevalence of stunting at 10 years. These findings may be explained by both biological and environmental factors such as differences in foetal growth [38,39], variation in susceptibility to insults during early development [40] as well as gender inequalities[41,42]. It is also important to consider the prospect of later sexual maturation among girls in our study sample relative to the WHO reference population, which could lead to an apparent inflation of the magnitude of growth deficiency relative to the boys. Although, some differences in growth retardation between the sexes was documented as early as 54 months[19].

Growth deficit in girls not only has immediate health consequences but is also associated with future reproductive outcomes [12] and potential trans-generational effects on their offspring. Pre-pregnancy maternal height is an indicator of long-term nutritional status and environmental exposures from foetal to adult life and is associated with the uterine volume, cephalo-pelvic disproportion and foetal growth restriction [43]. Our results show associations between maternal height, linear growth deficits and offspring stunting at ten years and are consistent with previous work from low- and middle-income countries stating maternal height as an important determinant of stunting [23,44,45]. Using cross-sectional data from 54 low- and middle-income countries, Özaltin and colleagues showed that increase in maternal height reduced the risk of child mortality, wasting and stunting[44].

The importance of pre-pregnancy and pregnancy nutrition is further supported by evidence from pooled analyses showing that growth faltering is being initiated during foetal life [13]. In our study season of conception was strongly associated with linear growth trajectories and stunting at ten years of age. In Bangladesh and in many other low- and middle-income countries levels of malnutrition show a seasonal variation [46] influenced by factors such as variation in food security, occurrence of infectious diseases and workload [47]. Season of conception is also linked to the season when the children were born and subsequently the season when they were measured at birthday follow-ups. However, a higher prevalence of stunting and lower HAZ scores among children conceived during the pre-monsoon season was documented at all ages, including the assessment at birth. Periods of relative food insecurity frequently occur in Bangladesh in May–June and October-November (before the two major rice harvests) [48]. Children conceived in the pre-monsoon period with relative food insecurity also enter into the third trimester during the second food insecurity period. The peak of diarrheal diseases occurring during the pre-monsoon season [49] may be an additional environmental factor that influences the health of mothers and foetuses [46].

Growth faltering is closely related to environmental and social factors such as frequent infections and inadequate nutrition and feeding practices [9]. Women are the primary caregivers and women’s education levels and position in household and society are associated with their ability to provide care [50,51]. During the last few decades, Bangladesh has made exceptional health achievements regarding reduced total fertility rate and child mortality, although levels of stunting still prevail as one of the highest in the world [52]. The achievements in health can partly be attributed to the progress in women’s empowerment and education [53]. Today the proportion of girls attending primary school is greater than that of boys [52]. Our results show us that higher maternal education level is associated with less stunted growth at ten years of age.

We have described longitudinal growth patterns in a rural setting in Bangladesh and shown that biological, social and environmental factors present at conception continue to be associated with childhood growth and the risk of being stunted at the age of ten. A lifecycle approach is needed for the prevention of impaired child growth, targeting women before and during pregnancy including continuous efforts to promote child growth during and beyond infancy.

## Supporting Information

S1 Table Baseline characteristics of mothers at 8 week of gestation and children at birth participating in the MINIMat trial, Bangladesh. Stratified for non-analyzed and analyzed children.
(PDF)

S2 Table Mean height for age Z-scores and mean difference in height from reference of children participating in the MINIMat trial, Bangaldesh, from birth to ten years. (PDF)

S3 Table Prevalence of stunting of children participating in the MINIMat trial, Bangladesh, from birth to ten years. (PDF)

S1 Data MINIMat data. Height for age scores for children participating in the MINIMat trial from birth to ten years of age, the final sample. N = 1054.
(CSV)
