# Supplementary material for: Stunted at 10 Years. Linear Growth Trajectories and Stunting from Birth to Pre-Adolescence in a Rural Bangladeshi Cohort
Source: PLoS One. 2016 Mar 2;11(3):e0149700. doi: 10.1371/journal.pone.0149700 (PMC4775024; doi:10.1371/journal.pone.0149700)
Supplement: S1 Table — Stratified for non-analyzed and analyzed children. (PDF) [file pone.0149700.s001.pdf]

S1 Table. Baseline characteristics of mothers at 8 week of gestation and children at birth participating in the MINIMat trial, Bangladesh. Stratified for non-analyzed and analyzed children.

CHARACTERISTICS		Children not analyzed[1] N=536		Children included in the analysis N=1054	
		%	n	%	n
Mothers					
Age					
	<20	19.8	106/536	14.0	148/1054
	20-29	53.9	289/536	56.5	595/1054
	≥30	26.3	141/536	29.5	311/1054
BMI at 8 week					
	<18,5	27.6	147/533	31.0	325/1049
	≥18,5	72.4	386/533	69.0	724/1049
Height					
	Shortest (<147.5 cm)	31.8	170/535	14.9	157/1052
	Average (≥147,5<152 cm)	32.7	175/535		
	Tallest (>152 cm)	35.5	190/535	85.1	895/1052
Education					
	No Education	29.1	156/536	38.3	404/1054
	1-5 years	70.9	380/536	61.7	650/1054
	> 5 years				
SES					
	Lowest	32.5	174/536	26.6	280/1054
	Middle	28.4	152/536	33.7	355/1054
	Highest	39.2	210/536	39.8	419/1054
Children					
Sex					
	Girl	47.6	255/536	48.7	541/1054
	Boy	52.4	281/536	51.3	513/1054
Season of conception					
	Winter (Nov-Feb)	32.8	176/536	33.5	353/1054
	Pre-monsoon (Mars-May)	29.7	159/536	29.5	311/1054
	Monsoon (Jun-Oct)	37.7	176/536	37.0	390/1054
SGA[2]		61.9	332/536	61,9	652/1054
LBW[3]		36.0	192/534	32.2	339/1053
Stunted[4]		18.3	98/536	16.1	170/1054
Preterm[5]		10.4	56/536	8.2	86/1054

[1] Children born between April 2002 and June 2003 not analyzed due to losses of follow up or missing data.
[2]Small for gestational age (Birth weight below reference for gestational week at birth (54)
[3]Low Birth Weight (<2500 g)
[4]Below 2 SD from WHO growth standards
[5]Born before 37 weeks of gestation
