# Supplementary material for: Stunted at 10 Years. Linear Growth Trajectories and Stunting from Birth to Pre-Adolescence in a Rural Bangladeshi Cohort
Source: PLoS One. 2016 Mar 2;11(3):e0149700. doi: 10.1371/journal.pone.0149700 (PMC4775024; doi:10.1371/journal.pone.0149700)
Supplement: S2 Table — (PDF) [file pone.0149700.s002.pdf]

S2 Table. Mean height for age scores and mean difference in height from reference of children participating in the MINIMat trial, Bangaldesh, from birth to ten years.

Age		Girls			Boys			Total	
**Months**		**Mean HAD[1] (SD)**	**Mean HAZ[2] (SD)**		**Mean HAD[1] (SD)**	**Mean HAZ[2] (SD)**		**Mean HAD[1] (SD)**	**Mean HAZ[2] (SD)**
0		-1.65 (2.09)	-0.89 (1.12)		-1.78 (2.23)	-0.94 (1.18)		-1.72 (2.17)	-0.91 (1.15)
1		-2.15 (2.10)	-1.10 (1.08)		-2.32 (2.25)	-1.19 (1.16)		-2.24 (2.18)	-1.15 (1.12)
2		-2.23(2.07)	-1.10 (1.02)		-2.47 (2.25)	-1.23 (1.13)		-2.35 (2.17)	-1.17 (1.08)
3		-2.41 (2.12)	-1.15 (1.01)		-2.67 (2.27)	-1.30 (1.11)		-2.54 (2.20)	-1.23 (1.06)
4		-2.47 (2.07)	-1.14 (0.96)		-2.70 (2.31)	-1.30 (1.01)		-2.59 (2.20)	-1.22 (1.04)
5		-2.58 (2.08)	-1.16 (0.94)		-2.70 (2.16)	-1.28 (1.02)		-2.64 (2.12)	-1.22 (0.98)
6		-2.75 (2.14)	-1.22 (0.95)		-2.99 (2.32)	-1.40 (1.09)		-2.88 (2.24)	-1.31 (1.02)
7		-2.90 (2.20)	-1.25 (0.95)		-3.22 (2.40)	-1.48 (1.03)		-3.06 (2.31)	-1.37 (1.03)
8		-3.11 (2.24)	-1.31 (0.95)		-3.30 (2.47)	-1.50 (1.12)		-3.21 (2.36)	-1.41 (1.04)
9		-3.30 (2.34)	-1.36 (0.97)		-3.48 (2.47)	-1.55 (1.10)		-3.39 (2.41)	-1.46 (1.04)
10		-3.56 (2.37)	-1.44 (0.96)		-3.72 (2.54)	-1.63 (1.11)		-3.64 (2.46)	-1.54 (1.04)
11		-3.79 (2.40)	-1.50 (0.95)		-4.00 (2.56)	-1.72 (1.11)		-3.90 (2.49)	-1.61 (1.04)
12		-3.98 (2.52)	-1.55 (0.98)		-4.17 (2.65)	-1.76 (1.11)		-4.08 (2.59)	-1.65 (1.06)
15		-4.87 (2.59)	-1.78 (0.95)		-5.00 (2.78)	-1.98 (1.10)		-4.94 (2.69)	-1.88 (1.03)
18		-5.46 (2.88)	-1.88 (0.99)		-5.53 (2.95)	-2.05 (1.09)		-5.50 (2.91)	-1.97 (1.05)
21		-6.07 (2.97)	-1.98 (0.97)		-6.14 (3.22)	-2.14 (1.12)		-6.11 (3.11)	-2.06 (1.05)
24		-6.61 (3.20)	-2.05 (0.99)		-6.59 (3.36)	-2.16 (1.10)		-6.60 (3.28)	-2.10 (1.05)
54		-7.53 (3.99)	-1.66 (0.88)		-6.85 (4.27)	-1.55 (0.97)		-7.18 (4.15)	-1.60 (0.93)
120		-9.54 (6.89)	-1.49 (1.08)		-8.78 (6.01)	-1.38 (0.94)		-9.15 (6.46)	-1.43(1.01)

[1] Height in cm from WHO growth reference median

[2]Height-for-age z-scores
