# Supplementary material for: Stunted at 10 Years. Linear Growth Trajectories and Stunting from Birth to Pre-Adolescence in a Rural Bangladeshi Cohort
Source: PLoS One. 2016 Mar 2;11(3):e0149700. doi: 10.1371/journal.pone.0149700 (PMC4775024; doi:10.1371/journal.pone.0149700)
Supplement: S3 Table — (PDF) [file pone.0149700.s003.pdf]

S3 Table. Prevalence of stunting of children participating in the MINIMat trial, Bangladesh, from birth to ten years.

Age		Girls			Boys			Total	
Months		n/N Stunted[1]	% Stunted[1]		n/N Stunted[1]	% Stunted[1]		n/N Stunted[1]	% Stunted[1]
0		81/513	15.8		89/541	16.5		170/1054	16.1
1		102/512	19.9		113/540	20.9		215/1052	20.4
2		94/513	18.3		133/541	24.6		227/1054	21.5
3		89/512	17.4		136/541	25.1		225/1053	21.4
4		85/513	16.6		133/541	24.6		218/1054	20.7
5		84/511	16.4		108/541	20.0		192/1052	18.3
6		96/513	18.7		152/541	28.1		248/1054	23.5
7		107/512	20.9		155/540	28.7		262/1052	24.9
8		112/512	21.9		179/541	33.1		291/1053	27.6
9		124/512	24.2		182/540	33.7		306/1052	29.1
10		135/512	26.4		191/539	35.4		326/1051	31.0
11		146/511	28.6		215/540	39.8		361/1051	34.3
12		155/509	30.5		215/540	39.8		370/1049	35.3
15		210/511	41.1		253/540	46.9		463/1051	44.1
18		236/512	46.1		284/541	52.5		520/1053	49.4
21		264/512	51.6		307/540	56.9		571/1052	54.3
24		260/513	50.7		295/541	54.5		555/1054	53.7
54		180/513	35.1		179/541	33.1		359/1054	34.1
120		166/513	32.4		142/541	26.2		308/1054	29.2

[1] Below minus 2 standard deviations from WHO growth reference median
